# The Janus face of uranium in toxicology

**DOI:** 10.1007/s00204-022-03223-3

**Published:** 2022-02-04

**Authors:** Hermann M. Bolt

**Affiliations:** Leibniz Research Centre on Working Environment and Human Factors (IfADo) Ardeystr. 67, 44139 Dortmund, Germany

The first publication on uranium in Archives of Toxicology (at that time: “*Fühner-Wieland’s Sammlung von Vergiftungsfällen*”) dates back to its second volume, when Woldrich and Bachem (1931) reported on the health status of uranium miners and workers in Joachimsthal/Bohemia (now: Jáchymov, Czech Republic), which were exposed to uranium and its radioactive decay products, especially radium and radon. Anemia and leukopenia were frequently observed, most pronounced in currently active uranium factory workers (7 out of 8 males with < 4 million erythrocytes per µL). Similar symptoms were reported at the same time in American females employed with radioactive decay products of uranium (Martland and Bachem 1931).

Uranium mining in Jáchymov was abandoned in 1964, but it was intensified on the other side of the border, in East Germany where from 1946 to 1990 more than 400,000 persons were employed by *Wismut AG* (a camouflage name, German word for bismuth), which mined more than 230,000 tons of uranium ore, mainly used for Soviet nuclear weapons. *Wismut* employees were exposed to a variety of occupational risk factors, primarily the inhalation of radon and exposure to its radioactive progeny elements. Recent studies confirm a marked excess mortality in *Wismut* underground miners due to silicosis/pneumoconiosis and lung cancer (Kreuzer et al 2021).

While the health consequences of radioactivity of uranium and its decay products have been known for decades, reasonable scientific interest in “conventional” toxicology of uranium has developed much later, and was accentuated after the year 2000. To a significant extent, this interest into the other side of uranium toxicity was triggered by reports and speculations on “Gulf War Illness”, which had raised international concern. U.S. and British forces used depleted uranium in armor-piercing rounds to disable enemy tanks during the Gulf and Balkan Wars, which led to speculations on possible associations between exposure to depleted uranium and adverse health outcomes among veterans and civilians (Marshall 2007; Shaki et al 2019; Surdyk et al 2021). A recent investigation of Parrish and Haley (2021) has resolved this question. The authors used a bio-kinetic model to predict the urinary concentration and uranium isotopic ratios for a range of inhalation exposures. Mass spectrometry was applied capable of detecting the predicted urinary depleted uranium to 154 individuals of a population-representative sample of U.S. veterans in whom Gulf War Illness had been determined by standard case definitions and depleted uranium inhalation exposures obtained by medical history. No difference was found in the ^238^U/^235^U ratio in veterans meeting the standard case definitions of Gulf War Illness versus control veterans, no differences by levels of depleted uranium inhalation exposure, and no ^236^U, associated with depleted uranium, was detected. These findings showed that even the highest likely levels of depleted uranium inhalation played no role in the development of Gulf War Illness, leaving exposure to aerosolized organophosphate compounds (pesticides and sarin nerve agent) as the more likely cause(s) of Gulf War Illness (Parrish and Haley 2021).

By contrast to adverse health effects caused by radioactivity, the kidneys are known as main toxicity target for systemically available uranium compounds, as well as the lungs after inhalation of poorly soluble uranium compounds (DFG 2015). Thus, during the last 15 years, publications in Archives of Toxicology dealt with mechanistic aspects of both nephrotoxicity (Goldman et al 2006; Zhu et al 2009) and lung toxicity (Periyakaruppan et al 2007, 2009) of uranium. But also other aspects were highlighted, namely effects of depleted uranium on CYP enzymes, with consequences on xenobiotic and vitamin D metabolism (Guéguen et al 2006, 2014; Tissandie et al 2006), and effects on the bone matrix (Tasat et al 2007; Pierrefit-Carle et al. 2017; Gritsaenko et al 2021).

There is an environmental impact of uranium toxicity, due to exposure via drinking water and food (Shaki et al 2019). In a recent review, Bjørklund et al (2020) pointed out that phosphate fertilization is an important source of contamination with uranium in agriculture, mainly due to contamination in the phosphate rock used for fertilizer manufacture. Therefore, long-term use of uranium-bearing fertilizers might substantially increase the concentration of uranium in fertilized soils. Hydro-geochemical aspects related to uranium-containing water sources are a new field related to public health (Bjørklund et al 2020).

Thus, uranium toxicity, as a matter of environmental concern, will continue as a subject of toxicological investigative efforts.

## References

[CR1] Bjørklund G, Semenova Y, Pivina L, Dadar M, Rahman MM, Aaseth J, Chirumbolo S (2020). Uranium in drinking water; a public health threat. Arch Toxicol.

[CR2] DFG [Deutsche Forschungsgemeinschaft] (2015) Uranium (natural and depleted) and its inorganic compounds (inhalable fraction) The MAK-Collection Part I, MAK Value Documentations 2015: 1-22. 10.1002/3527600418.mb744061e5315

[CR3] Goldman M, Yaari A, Doshnitzki R, Cohen-Luria R, Moran A (2006). Nephrotoxicity of uranyl acetate: effect on rat kidney brush border membrane vesicles. Arch Toxicol.

[CR4] Gritsaenko T, Pierrefite-Carle V, Creff G (2021). Low doses of uranium and osteoclastic bone resorption: key reciprocal effects evidenced using new in vitro biomimetic models of bone matrix. Arch Toxicol.

[CR5] Guéguen Y, Souidi M, Baudelin C, Dudoignon N, Grison S, Dublineau I, Marquette C, Voisin P, Gourmelon P, Aigueperse J (2006). Short-term hepatic effects of depleted uranium on xenobiotic and bile acid metabolizing cytochrome P450 enzymes in the rat. Arch Toxicol.

[CR6] Guéguen Y, Rouas C, Minin A, Manens L, Stefani J, Delissen O, Grison S, Dublineau I (2014). Molecular, cellular, and tissue impact of depleted uranium on xenobiotic-metabolizing enzymes. Arch Toxicol.

[CR7] Kreuzer M, Deffner V, Schnelzer M, Fenske M (2021). Mortality in underground miners in a former uranium ore mine. Dtsch Ärztebl Int.

[CR8] Marshall AC (2007). Gulf war depleted uranium risks. J Expo Sci Environ Epidemiol.

[CR9] Martland HS, Bachem C (1931). Radium-Vergiftungen, chronische, gewerbliche, in Amerika. Vergiftungsfälle (Arch Toxicol).

[CR10] Parrish RR, Haley RW (2021). Resolving whether inhalation of depleted uranium contributed to Gulf War Illness using high-sensitivity mass spectrometry. Natureportfolio Sci Rep.

[CR11] Periyakaruppan A, Kumar F, Sarkar S, Sharma CS, Ramesh GT (2007). Uranium induces oxidative stress in lung epithelial cells. Arch Toxicol.

[CR12] Periyakaruppan A, Sarkar S, Ravichandran P, Sadanandan B, Sharma CS, Ramesh V, Hall JS, Thomas R, Wilson BL, Ramesh GT (2009). Uranium induces apoptosis in lung epithelial cells. Arch Toxicol.

[CR13] Pierrefite-Carle V, Santucci-Darmanin S, Breuil V (2017). Effect of natural uranium on the UMR-106 oesteoblastic cell line: impairment of the autophagic process as an underlying mechanism of uranium toxicity. Arch Toxicol.

[CR14] Shaki F, Zamani E, Arjmand A, Pourahmad J (2019). A review on toxicodynamics of depleted uranium. Iran J Pharm Res.

[CR15] Surdyk S, Itani M, La-Lobaidy M, Kahale LA, Farha A, Dewachi O, Aki EA, Habib RR (2021). Weaponised uranium and adverse health outcome in Iraq: a systematic review. BMJ Glob Health.

[CR16] Tasat DR, Orona NS, Mandalunis PM, Cabrini RL, Ubios AM (2007). Ultrastructural and metabolic changes in osteoblasts exposed to uranyl nitrate. Arch Toxicol.

[CR17] Tissandie E, Guéguen Y, Lobaccaro JMA, Paquet E, Aigueperse J, Souidi M (2006). Effects of depleted uranium after short-term exposure on vitamin D metabolism in rat. Arch Toxicol.

[CR18] Woldrich A, Bachem C (1931). Radium-Vergiftungen, chronische, gewerbliche, in Joachimsthal. Vergiftungsfälle (Arch Toxicol).

[CR19] Zhu G, Xiang X, Chen X, Wang L, Hu H, Weng S (2009). Renal dysfunction induced by long-term exposure to depleted uranium in rats. Arch Toxicol.

